# Brain tumor with an *ATXN1-NUTM1* fusion gene expands the histologic spectrum of *NUTM1*-rearranged neoplasia

**DOI:** 10.1186/s40478-019-0870-8

**Published:** 2019-12-30

**Authors:** Aurore Siegfried, Julien Masliah-Planchon, Franck-Emmanuel Roux, Delphine Larrieu-Ciron, Gaelle Pierron, Yvan Nicaise, Marion Gambart, Isabelle Catalaa, Sarah Péricart, Charlotte Dubucs, Badreddine Mohand-Oumoussa, Franck Tirode, Franck Bourdeaut, Emmanuelle Uro-Coste

**Affiliations:** 1 Departments of Pathology, Neurology, Neurosurgery, Radiology and Pediatric Oncology, Toulouse University Hospital, Toulouse, France; 2 INSERM U1037, Cancer Research Center of Toulouse (CRCT), Toulouse, France; 3 Departments of Genetics and of Oncopediatry and Young Adults, Curie Institute, Paris, France; 4 INSERM U830, Laboratory of Translational Research in Pediatric Oncology, SIREDO pediatric oncology center, Curie Institute, Paris, France; 5 Department of Somatic Genetics, Curie Institute, Paris, France; 6 Plateforme Post-génomique P3S, Faculté de Médecine Pierre et Marie Curie, Paris, France; 7 INSERM 1052, CNRS 5286, Cancer Research Center of Lyon, Centre Léon Bérard, Claude Bernard Lyon 1 University, Lyon, France

**Keywords:** *NUTM1*, *ATXN1*, *NUTM1*-rearranged neoplasia, RNA sequencing, DNA methylation-based classification, Central nervous system, Oncogenic gene fusions, CIC-ATXN1-ATXN1L axis

We report a novel *ATXN1-NUTM1* gene fusion in a primitive brain tumor (Fig 1a). A 21-year-old woman was seen in an emergency department for symptoms of increased intracranial pressure, visual disturbance and right hemiparesis. She reported unusual headaches for the past 3 weeks. MRI showed a frontal tumor with intratumoral hemorrhage (Fig. 1b). The entire tumor was surgically removed. The patient did not receive any additional treatment. 16 months after surgery, the patient was symptom-free and MRI showed no recurrence of the tumor.

**Fig. 1:**
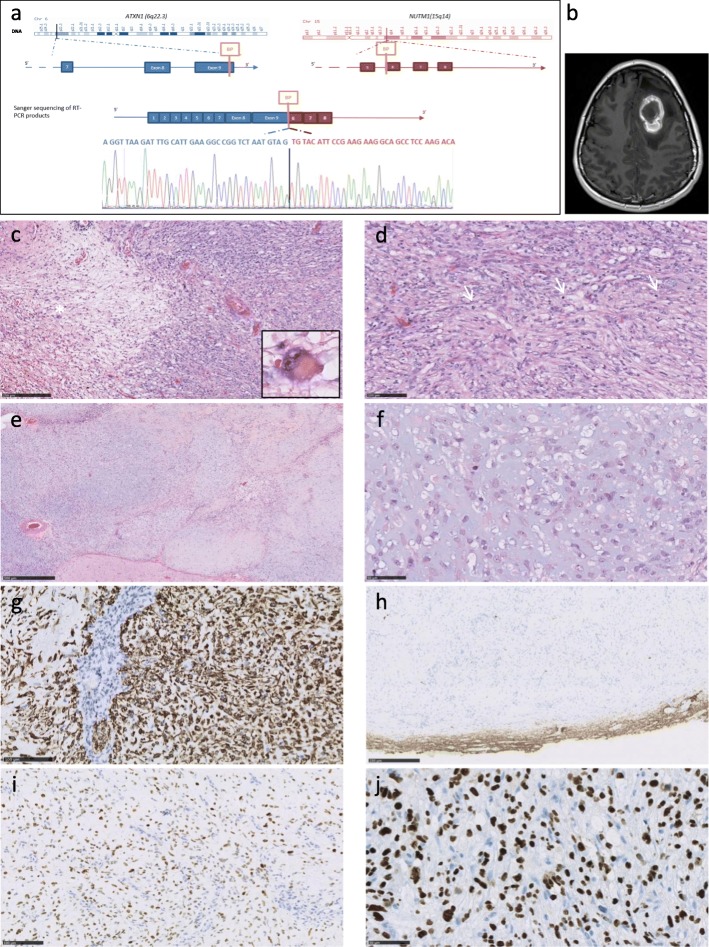
*ATXN1-NUTM1* gene fusion, confirmed by RT-PCR and Sanger sequencing (**a**). MRI identified a frontal mass. Enhancement after contrast injection (T1) (**b**). Representative histopathology. On the left, loose area with neuron-like tumor cells (*detail). On the right, increase in cell density (**c**). Fascicular architecture with three mitoses (arrows) (**d**). Chondroid-like, myxoid and hyalinized areas were observed (**e**). Undifferentiated cells with large nucleoli in a chondromyxoid background (**f**). Strong GFAP staining was observed. Tumor showed vascular proliferation (**g**). Neurofilament staining circumscribed the tumor mass with no significant staining within the tumor (**h**). p53 accumulated in tumor nuclei (**i**). Anti-NUT antibody staining showing homogeneous intranuclear expression (**j**)

Histological features were characterized by a fascicular architectural pattern and chondro-myxoid areas (Fig. 1c, d, e, f). Neuron-like tumor cells were apparent (Fig. 1c). Mitotic activity was overall low but increased in some foci (Fig. 1d). Strong GFAP staining led to an initial diagnosis of an unclassified glioneuronal tumor in spite of olig2 and PS100 negativity (Fig. 1g). Microscopically, the tumor was well circumscribed (Fig. 1h). p53 was accumulated (Fig. 1i). CD56 was strongly expressed. TTF1, chromogranin, synaptophysin, CD34, p63, CK5/6 and smooth muscle actin were negative. ATRX, INI1 and BRG1 expression was maintained. Using the Heidelberg DNA methylation-based CNS tumor classifier, no class prediction was obtained with a greater than ≥0.9 confidence threshold [1]. The closest entity was the CNS Ewing Family Tumor *CIC* group with a score of 0.235 (Additional file 1: Table S1) (Case methylation data: http://www.ncbi.nlm.nih.gov/geo; GSE138550). This tumor group is associated to the *CIC-NUTM1* gene fusion [6]. We observed strong homogeneous nuclear staining with an anti-NUT antibody, suggesting the presence of a *CIC-NUTM1* fusion (Fig. 1j). RNA sequencing using the Illumina TruSight RNA Fusion panel and Manta for fusion calling revealed a novel *ATXN1-NUTM1* fusion. A *CIC-NUTM1* fusion was not detected. *ETV4* was overexpressed as in CIC-fused sarcomas [4, 6]. No pathogenic variants were observed in tumor DNA using a 571-gene targeted sequencing panel (Additional file 2: Table S2).

The fusion gene transcript encompassed almost all of the *ATXN1* coding sequence and the entire exon 6, 7 and 8 regions of *NUTM1*. The most common *NUTM1* breakpoints map between exon 1 and 2, but breakpoints at the distal end of exon 5 have also been described in some *CIC-NUTM1* sarcomas [4].

Initially associated with NUT midline carcinomas, *NUTM1* fusions have now been described in a broad spectrum of tumors ranging from carcinoma to sarcoma and leukemia [2, 3, 7]. The most common fusion partner gene in carcinoma and sarcoma is *BRD4* followed by *BRD3* and *NSD3*. Various new partners have been recently described [2, 3, 5]. The prognosis of these tumors is generally poor, although NUT-associated leukemias appear to be associated with a better prognosis and *YAP1-NUTM1* is associated with benign skin adnexal gland tumors [3, 5].

*CIC* rearranged sarcomas are often fused to *DUX4* and less frequently to *NUTM1* [4, 7]. All *CIC* re-arranged tumors irrespective of their location or their fusion partner gene share the same transcriptomic profile defining a molecular subgroup distinct from NUT carcinoma [4, 7]. Interestingly, *ATXN1* codes for ataxin1 which forms a transcriptional repressor complex with CIC. They are both part of the CIC-ATXN1-ATXN1L mitotic cell cycle regulator axis [8]. Excluding *CIC-NUTM1* fused tumors, only one *NUTM1* rearranged brain tumor has been previously reported, namely a cytokeratin negative *BRD4-NUTM1* PNET-like parietal lobe tumor in a 3-year old boy with GFAP and synaptophysin positivity. On methylation profiling, this neoplasm did not cluster with tumors of the CNS Ewing Family Tumor *CIC* group [2].

Myxoid and chondroid differentiation has been reported in *NUTM1*-rearranged sarcomas but is unusual in primary glioneuronal tumors. Whether the strong GFAP positivity of our specific case is indicative of a glial tumor or of a sarcoma with myoepithelial differentiation cannot be assessed due to the lack of positive staining and specificity for other markers tested. GFAP positivity has been described in 3 out of 4 *NUTM1* rearranged soft tissue or visceral sarcomas, this is in contrast to the CNS Ewing Family Tumor *CIC* group which fails to express any differentiation markers [2, 6]. We recommend performing NUT immunohistochemistry followed by RNA sequencing to identify any potential *NUTM1* fusion partner genes in GFAP+/olig2- unclassified glioma, particularly those with myxoid and/or chondroid features. The *ATXN1*-*NUTM1* fusion gene may define a novel group of rare primary brain tumors. The prognostic influence of *NUTM1* fusion partners and the brain localization of *NUTM1*-rearranged tumors warrant further investigation.

## Supplementary information

**Additional file 1: Table S1.** Results of the Heidelberg DNA methylation-based CNS tumor classifier (entities and scores).

**Additional file 2: Table S2.** List of the 517 childhood cancer genes in the dragon targeted gene sequencing panel (Illumina_TruSeq Custom Amplicon).
